# Infertility-related stress and marital intimacy among infertile couples undergoing in vitro fertilization treatment in Western Iran: A cross-sectional study

**DOI:** 10.1016/j.eurox.2025.100384

**Published:** 2025-03-25

**Authors:** Sara Abdoli, Salman Khazaei, Faezeh Fazli, Shamim Pilehvari, Ensiyeh jenabi

**Affiliations:** a Student Research Committee, Hamadan University of Medical Sciences, Hamadan, Iran; b Malayer School of Medical Sciences, Hamadan University of Medical Sciences, Hamadan, Iran; c Research Center for Health Sciences, Hamadan University of Medical Sciences, Hamadan, Iran; d Fertility and Infertility Research Center, Hamadan University of Medical Sciences, Hamadan, Iran; e Mother and Child Care Research Center, Hamadan University of Medical Sciences, Hamadan, Iran

**Keywords:** Marital intimacy, Stress, Infertility, In vitro fertilization

## Abstract

**Background:**

Given the fundamental role of fertility and childbearing in Iranian families, along with its cultural and social dimensions, and considering the importance of paying attention to marital issues among infertile couples, despite the limited and contradictory studies in this area, the present research aimed to Study marital intimacy and infertility stress in infertile couples undergoing in Vitro Fertilization (IVF) treatment in the west of Iran.

**Methods:**

In 2023, a cross-sectional study from 1 September until December was undertaken on 117 couples at an Infertility Center in Hamadan City, Iran. Measurement tools were demographic characteristics, Newton's infertility stress questionnaire and the marital intimacy questionnaire. A convenience sampling method was utilized to recruit a total of 117 eligible women, each part of a couple, for the study. All participants completed the questionnaires of demographic characteristics, newton's infertility stress and the marital intimacy as required. Data analysis was carried out using Stata-17 software, with significance set at a level below 0.05.

**Results:**

In the present study the number of 234 cases was investigated. There wasn't a significant difference observed in infertility-related stress domains by gender (P > 0.05).Significant direct correlations were observed between infertility-related stress and age (r = 0.55, P < 0.001), duration of marriage(r = 0.72, P < 0.001), duration of infertility (r = 0.64, P < 0.001), and duration of treatment (r = 0.67, P < 0.001). Conversely, marital intimacy exhibited significant inverse correlations with these variables (P < 0.001). Additionally, a significant negative correlation was found between marital intimacy and infertility-related stress (r = -0.67, P < 0.001).

**Conclusion:**

The present study has shown that the mean score of marital intimacy among women is significantly higher than men. Additionally, there is a significant negative correlation between marital intimacy and infertility-related stress. Considering the importance of mental health in infertile couples and its potential impact on the outcome of IVF treatment, it appears that supportive interventions and counseling sessions to increase marital intimacy in infertile couples undergoing IVF treatment.

## Introduction

Infertility, as a bitter experience of life, leads to social and psychological stress and reduces satisfaction with life. Worldwide, almost 15 percent of couples suffer from infertility. The prevalence of infertility varies between 5.3 and 30 percent in different countries [1]. In Iran, the prevalence of infertility ranges from 3.10 to 9.24 percent [2], and this variability is likely due to the study population, definitions of infertility, and estimation methods [1].

Infertility can be one of the most challenging hurdles that a couple may face and can impact many aspects of a relationship. It is undeniable that infertility greatly affects both mental and physical health [14]. Scientific research indicates that infertility is often a profoundly distressing experience, a reality that worsens with the significant changes it brings to couples' relationships [15], [16]. Shame, embarrassment, despair, fear, and financial pressure can all lead to significant rifts between partners [17]. Feelings of guilt and blame may also arise, especially if one partner is identified as the primary cause of infertility. Additionally, the infertile partner may fear abandonment by the other [18]. Most infertile couples experience stress and tension during the treatment process, which can have detrimental effects on their relationships [4]. Marital intimacy is a process that develops over the course of life and affects the health of the couple, as well as being a fundamental factor in the strength and preservation of marital bonds [3]. Marital intimacy is a state in which, most of the time, spouses feel happiness and satisfaction with each other, created through mutual affection, caring for each other, acceptance, understanding each other, and satisfying needs, including sexual needs [4].

Infertility and its treatment, as a crisis in shared life, not only create psychological problems but can also act as a powerful blow against marital relationships [5]. This issue becomes even more important when we realize that marital intimacy is the most important source of support during infertility treatment [6]. Since infertility affects couples as a unit, it is necessary to make extra efforts to establish appropriate communication between spouses [7].

Levels of stress and marital intimacy may vary between men and women. In a study examining the level of infertility stress among infertile couples, it was found that men experience less stress compared to women, and women are more affected by the psychological effects of infertility than men [8]. Additionally, scientific research has shown that the level of marital intimacy is higher in fertile couples compared to infertile couples [9[.

So far, no study has been conducted to compare marital intimacy and infertility stress in infertile couples in Iran. Considering the fundamental role of fertility and childbearing in Iranian families, along with its cultural and social dimensions, and considering the importance of paying attention to marital issues among infertile couples, the present research aimed to Study marital intimacy and infertility stress in infertile couples undergoing in Vitro Fertilization (IVF) treatment attending the Fatemiyeh Infertility Center in the west of Iran.

## Materials and methods

In 2023, a cross-sectional study, from 1 September until December was undertaken at Fatemeieh Infertility Center in Hamadan City, located in western Iran. Prior to their participation, couples provided informed consent. The study received approval from the Ethics Committee of Hamadan University of Medical Sciences under registration ID IR.UMSHA.REC.1402.430.

The inclusion criteria encompassed individuals who were married and living stably with their spouse (no divorce), diagnosed with primary infertility confirmed by a physician (male factor, female factor, or unknown), husbands without a history of polygamy, absence of mental illnesses as per self-report, no severe family conflicts, no smoking or drug addiction, no recent adverse events in the last month, and no history of psychiatric hospitalization. Exclusion criteria involved incomplete questionnaire responses. Furthermore, couples aiding each other in completing the questionnaires were also excluded from the study.

Despite an exhaustive search across various sources and texts, no existing study was found that concurrently assessed the correlation between marital intimacy and infertility -related stress. Consequently, researchers proceeded to conduct a pilot study involving 20 participants, revealing a correlation coefficient of 0.21 between the two variables. Based on a desired power of 90 % and a type 1 error level of 0.05, the sample size was calculated using the following formula, resulting in a requirement of 234 individuals (117 couples).

$$n\geq {\left(\frac{Z_{1-\alpha /2}+Z_{1-\beta }}{\frac{1}{2}\log_{e}\frac{1+r}{1-r}}\right)}^{2}+3$$

Demographic characteristics included the age of the couples, occupation, education level, income, duration of infertility, duration of treatment and duration of marriage.

Newton's infertility stress questionnaire: The multi-dimensional instrument was developed by Newton et al. in 1999 at the London Health Sciences Center. This questionnaire comprises 46 items and evaluates the concerns of infertile individuals across five subscales: social concerns (10 questions), sexual concerns (8 questions), relationship concern (10 questions), rejection of childfree lifestyle (8 questions), and need for parenthood (10 questions). Responses are recorded using a Likert scale ranging from 1 (Completely disagree) to 6 (Completely agree)[10]. The total score ranges from 46 to 276, with higher scores indicating greater infertility-related stress. In the research of Newton et al. (1999), the validity and reliability of this tool has been investigated. The factor analysis method was used to check the validity and the five subscales of this questionnaire were confirmed. All five scales, as well as the composite total scale, demonstrated moderate to high reliability (internal consistency) as measured by Cronbach's alpha: Social Concern = 0.87, Sexual Concern = 0.77, Relationship Concern = 0.82, Rejection of Childfree Lifestyle = 0.80, Need for Parenthood = 0.84, and Global Stress = 0.93[10]. Alizadeh et al., conducted psychometric testing of this questionnaire among 30 infertile individuals in Isfahan city, Iran, in 2005[11].In the study, face validity was assessed, with the translated questionnaire reviewed and approved by seven expert professors in the field. The reliability of the questionnaire was evaluated using Cronbach's alpha, yielding values of 0.78 for social issues, 0.77 for sex, 0.78 for relationship, 0.75 for lifestyle without children, 0.84 for the need to be a parent, and an overall score of 0.91[11].

The marital intimacy questionnaire: The Walker-Thompson Intimacy Questionnaire, comprising 17 questions, aims to gauge the level of affection and intimacy among married couples. Developed by Walker and Thompson [12],this scale is a component of a broader instrument encompassing various dimensions of intimacy; however, it can also be utilized independently for intimacy assessment. In the intimacy scale, the subject’s score is calculated by summing the scores of all questions and dividing by 17. Scores range from 1 to 7, with a higher score indicating greater intimacy. The classification of scores is as follows: a score between 17 and 34 indicates a low level of intimacy in life, a score between 34 and 85 indicates an average level, and a score above 85 indicates a high level of intimacy. Walker and Thompson (1983) reported that scale reliability, measured by Cronbach's alpha, ranged from 0.91 to 0.97[12]. In Iran, Etemadi (2005) reported Cronbach's alpha coefficients for the scale as 0.96 and 0.85, respectively [13].Infertile couples meeting the eligibility criteria were included in the study through convenience sampling. All questionnaires were completed with the couples' consent at the Fatemeieh Infertility Center in Hamadan City.

The analysis of the data involved utilizing the mean and standard deviation to describe quantitative variables, while frequency and percentage were employed for qualitative variables. Student’s *t*-test and analysis of variance (ANOVA) were used to compare the infertility-related stress and marital intimacy according categorical variables. Bivariate Pearson correlations were conducted to examine the association between marital intimacy and infertility-related stress. Data analysis was carried out using Stata-17 software, with significance set at a level below 0.05.

## Results

In the present study the number of 234 couples (117 man and 117 women) was investigated. All participants completed questionnaires. The mean age of them was 36.21 ± 3.97 years [37.99 ± 3.82 years in men and 34.44 ± 3.28 years in women (P < 0.001)]. Duration of marriage, infertility and treatment of them were 2.52 ± 1.02, 2.32 ± 0.79, and 2.3 ± 0.87 years, respectively (Table 1).

**Table 1:** The mean of continuous variables in the investigated patients.

**Variable**	**Mean ± SD**	**Range**
Age (year)	37.99 ± 3.82	25–47
Duration of marriage (year)	2.52 ± 1.02	1–5
Duration of infertility (year)	2.32 ± 0.79	1–4
Duration of treatment (year)	2.3 ± 0.87	1–4

Table 2 presents a comparison of infertility-related stress and marital intimacy domains across genders. It's evident that there wasn't a significant difference observed in infertility-related stress domains by gender (P > 0.05). However, the mean score for marital intimacy was notably higher among women (37.7 ± 13.6 vs. 32.45 ± 11.77, P = 0.002).

**Table 2:** Comparison of infertility stress andmarital intimacy domains by gender.

**Variable**		**Men Mean ± SD**	**Women Mean ± SD**	**P.Value**
Infertility-related stress	Social concern	44.84 ± 12.48	44.43 ± 10.92	0.79
	Sexual concern	36.26 ± 10.49	35.31 ± 9.35	0.47
	Relationship concern	44.67 ± 12.59	43.2 ± 11.1	0.35
	Rejection of childfree lifestyle	36.38 ± 9.86	35.01 ± 9.05	0.27
	Need for parenthood	44.8 ± 12.91	43.48 ± 11.73	0.41
	Total infertility	206.95 ± 57.06	201.08 ± 50.73	0.41
Marital Intimacy		32.45 ± 11.77	37.7 ± 13.6	**0.002**

Statistical test: Student’s *t*-test

As shown in Table 3 the marital intimacy was significantly higher in women (P = 0.002), academic education (P < 0.001), employee cases (P < 0.001), moderate economic (P < 0.001), have insurance (P = 0.008), lived in organizational house (P < 0.001), and number of IVF failure (none and one) (P < 0.001). Moreover, infertility-related stress was significantly higher in patients with high school education (P < 0.001), workless occupation (P < 0.001), poor economic situation (P < 0.001), not having insurance (P < 0.001), living with relatives (P < 0.001), three IVF failure (P < 0.001), and having consanguineous marriage(P < 0.001).Significant direct correlations were observed between infertility-related stress and the age of the couples(r = 0.55, P < 0.001), duration of marriage (r = 0.72, P < 0.001), duration of infertility (r = 0.64, P < 0.001), and duration of treatment (r = 0.67, P < 0.001) (Table 4). Conversely, marital intimacy exhibited significant inverse correlations with these variables (P < 0.001). Additionally, a significant negative correlation was found between marital intimacy and infertility-related stress (r = -0.67, P < 0.001) (Table 4).

**Table 3:** Comparison of infertility-related stress andmarital intimacy according categorical variables.

**Variable**		**N (%)**	**Marital Intimacy**		**Infertility-related stress**	
			Mean± SD	P.Value	Mean± SD	P.Value
Gender	Man	117 (50)	32.45 ± 11.77	**0.002**	206.95 ± 57.06	0.41
	Woman	117 (50)	37.7 ± 13.6		201.08 ± 50.57	
Education	Primary	27 (11.54)	31.33 ± 8.23	**< 0.001**		**< 0.001**
	High school	68 (29.05)	29.5 ± 6.33			
	Diploma	102 (43.59)	36.03 ± 13.82			
	Academic	37 (15.81)	45.43 ± 15.67			
Occupation	Housewife	104 (44.44)	35.7 ± 12.22	**< 0.001**	212.85 ± 40.24	**< 0.001**
	Employee	26 (11.11)	52.69 ± 14.34		109.96 ± 30.8	
	Workless	12 (5.13)	26.33 ± 6.4		228.58 ± 23.57	
	Self-employed	69 (29.49)	31.54 ± 9.75		212 ± 53.73	
	Manual worker	23 (9.83)	27.52 ± 4.58		234.09 ± 23.38	
Economic situation	Poor	52 (22.22)	29.87 ± 6.35	**< 0.001**	229.19 ± 21.61	**< 0.001**
	Moderate	182 (77.78)	36.57 ± 13.95		196.79 ± 58.1	
Insurance status	Yes	196 (83.76)	36.06 ± 13.62	**0.008**	199.95 ± 54.44	**0.009**
	No	38 (16.24)	30.03 ± 6.91		224.94 ± 19.29	
Housing situation	Mortgage or lease	146 (62.39)	31.16 ± 7.83	**< 0.001**	221.73 ± 35.71	**< 0.001**
	private house	36 (15.38)	49.61 ± 17.21		132.75 ± 54.45	
	Organizational house	14 (5.98)	51.93 ± 17.48		127.93 ± 49.32	
	Living with relatives	38 (16.24)	30.16 ± 5.57		232.03 ± 20.55	
Number of IVF failure	None	30.16 ± 5.57	38 (16.24)	**< 0.001**	106 ± 8.37	**< 0.001**
	One	63.5 ± 24.43	6 (2.56)		115.03 ± 33.18	
	Two	51.75 ± 11.8	28 (11.97)		189.98 ± 66.35	
	Three	40.67 ± 13.82	42 (17.95)		234.36 ± 27.7	
	4 or more	32.74 ± 6.12	74 (31.62)		221.36 ± 18.34	
Cause of infertility	Female factor	35.75 ± 12.9	88 (37.61)	0.63	206.74 ± 54.87	0.77
	Male factor	35.54 ± 14.34	66 (28.21)		200.42 ± 55.6	
	Both	33.95 ± 11.87	80 (34.19)		204.04 ± 51.86	
consanguineous marriage	Yes	33.91 ± 13.88	46 (19.66)	0.5	231.39 ± 36.3	**< 0.001**
	No	35.36 ± 12.74	188 (80.34)		197.29 ± 55.44	

Statistical test:Student’s *t*-test and analysis of variance (ANOVA)

**Table 4:** Correlation between infertility-related stress, marital intimacy domains and other continuous variables.

Variable		Marital Intimacy	Infertility-related stress	Age	Duration of marriage	Duration of infertility	Duration of treatment
Marital Intimacy	r	1					
	P.Value	-					
Infertility-related stress	r	−0.67	1				
	P.Value	< 0.001	-				
Age	r	−0.58	0.55	1			
	P.Value	< 0.001	< 0.001	-			
Duration of marriage	r	−0.61	0.72	0.56	1		
	P.Value	< 0.001	< 0.001	< 0.001	-		
Duration of infertility	r	−0.56	0.64	0.52	0.87	1	
	P.Value	< 0.001	< 0.001	< 0.001	< 0.001	-	
Duration of treatment	r	−0.54	0.67	0.51	0.88	0.91	1
	P.Value	< 0.001	< 0.001	< 0.001	< 0.001	< 0.001	-

## Discussion

In the current study, 117 couples undergoing IVF treatment were examined. The results indicated that the mean score of marital intimacy among women was significantly higher than that of men. However, no significant difference was observed in infertility-related stress based on gender. The current study demonstrated a significant negative correlation between marital intimacy and infertility-related stress among couples. Marital intimacy was significantly higher among women with academic education, office job occupations, moderate economic status, having insurance, and fewer IVF failures.

These findings reported a significant increase in marital intimacy was observed with a decrease in infertility stress among couples. Rezaei et al., showed a positive and significant correlation between infertility stress and the quality of marital relationship[14]. In other words, as infertility stress increased, the quality of marital relationship among infertile couples improved[14]. In a study comparing fertile and infertile women, the results showed that marital intimacy was significantly higher in fertile women than in those who were still infertile for any reason. It can be argued that fertility and having children contribute to greater warmth and intimacy in marital relationships [15]. In another study, perceived marital intimacy in normal couples was higher than in infertile couples. Therefore, infertility and its medical treatment have detrimental effects on mental and physical health, especially on the sexual life and intimacy of infertile women [9]. The aforementioned studies are relevant to the present research. It can be explained that infertile women experience signs of crisis, depression, mourning, loss of control, high levels of anxiety, and sexual dysfunction. If they blame themselves for infertility and have an undesirable perception of their physical condition, they show greater vulnerability to the resulting stress, leading to significant strain between them and their spouses. Consequently, they receive less support and empathy from their partners[16]. However, a study conducted to investigate marital intimacy in infertile women in northern Iran showed that 88.5 % of infertile women had good marital intimacy, which contradicts the findings of the present study[17].

Based on the findings published in the study by Amini et al. (2020), it has been determined that overall stress and the stress of the need for parenthood are higher in infertile women compared to their spouses. In fact, women facing infertility exhibit reactions such as anxiety, stress, depression, and grief. Among the most significant causes of stress in these women are fear of divorce and a sense of losing their ability to become mothers. It is a reality that since pregnancy and the maternal role are specific to women, societal expectations of women are higher than men and the fear of social stigma of infertility is higher in women [8].

In the present study, marital intimacy significantly decreased with increasing age and duration of marriage which is consistent with the findings of Arefi et al. (2016)[15]. In other words, as the duration of marriage and marital life increases, the intimacy and warmth in the relationship decrease[14].

This research will help spouses recognize their communication problems, identify the root causes of their differences so they can have positive and appropriate interactions with each other, express themselves in a more desirable manner, and ultimately impact the level of intimacy between them, especially sexual intimacy. It is recommended that in future research, targeted interventions in relationship adjustment and management for spouses be designed, and the outcome of these interventions be evaluated in improving family intimacy.

Some limitations of the current study include the self-report data collection method, which may lead to biases associated with this type of data. Another limitation of this study could be the stress and concerns related to infertility treatment, which might affect how respondents answer the marital intimacy questionnaire.

## Conclusion

The present study has shown that the mean score of marital intimacy among women is significantly higher than men. Additionally, there is a significant negative correlation between marital intimacy and infertility-related stress. However, marital intimacy acts as a protective factor, reducing infertility-related stress. In addition, the study highlights that infertility-related stress is influenced by factors such as age, duration of marriage, duration of infertility, and duration of treatment, with stress levels increasing as these factors increase.

Considering the importance of mental health in infertile couples and its potential impact on the outcome of IVF treatment, it appears that supportive interventions and counseling sessions to increase marital intimacy, enhance cooperation and support between spouses, and address inflexibility in couples particularly for individuals with longer durations of infertility or treatment, may help mitigate the stress associated with infertility could significantly improve the psychological status and marital relationship of infertile couples undergoing IVF treatment, thereby enhancing treatment response. It is hoped that the results of this study can be used to develop comprehensive health and treatment programs and benefit from them to promote mental health and adopt healthy health behaviors.

## CRediT authorship contribution statement

**abdoli sara:** Writing – review & editing, Writing – original draft, Visualization, Project administration, Methodology, Investigation, Formal analysis, Data curation, Conceptualization. **Khazaei Salman:** Writing – review & editing, Writing – original draft, Validation, Software, Methodology, Formal analysis. **Fazli Faezeh:** Writing – review & editing, Writing – original draft, Project administration, Methodology, Investigation, Data curation. **Pilehvari Shamim:** Writing – review & editing, Writing – original draft, Visualization, Project administration. **Jenabi Ensiyeh:** Writing – review & editing, Writing – original draft, Visualization, Validation, Supervision, Software, Resources, Project administration, Methodology, Investigation, Formal analysis, Data curation, Conceptualization.

## Declaration of Competing Interest

The authors declare that they have no known competing financial interests or personal relationships that could have appeared to influence the work reported in this article.
